# Sex Hormones and Gender Influence the Expression of Markers of Regulatory T Cells in SLE Patients

**DOI:** 10.3389/fimmu.2021.619268

**Published:** 2021-03-03

**Authors:** Ram P. Singh, David S. Bischoff

**Affiliations:** ^1^Research Service, Veteran Administration Greater Los Angeles Healthcare System, Los Angeles, CA, United States; ^2^David Geffen School of Medicine, University of California, Los Angeles, Los Angeles, CA, United States

**Keywords:** sex hormones, gender, regulatory T cells, systemic lupus erythematosus, 17β-estradiol

## Abstract

Regulatory T cells have been implicated in the regulation and maintenance of immune homeostasis. Whether gender and sex hormones differentially influence the expression and function of regulatory T cell phenotype and their influence on FoxP3 expression remains obscure. We provide evidence in this study that the number and percent of human regulatory T cells (T_regs_) expressing CD4^+^ and CD8^+^ are significantly reduced in healthy females compared to healthy males. In addition, both CD4^+^CD25^+hi^ and CD8^+^CD25^+hi^ subsets in healthy males have a 2-3 fold increase in FoxP3 mRNA expression compared to healthy females. Female SLE patients, compared to healthy women, have elevated plasma levels of estradiol and decreased levels of testosterone. Higher levels of testosterone correlate with higher expression of FoxP3 in CD4^+^CD25^hi^CD127^low^ putative T_regs_ in women with SLE. Incubation of CD4^+^ regulatory T cells with 17β-estradiol at physiological levels generally decreased FoxP3 expression in females with SLE. These data suggest that females may be more susceptible than males to SLE and other autoimmune diseases in part because they have fewer T_regs_ and reduced FoxP3 expression within those cells due to normal E2 levels which suppress FoxP3 expression. In addition, low levels of plasma testosterone in women may further reduce the ability of the T_regs_ to express FoxP3. These data suggest that gender and sex hormones can influence susceptibility to SLE via effects on regulatory T cells and FoxP3 expression.

## Introduction

Many autoimmune diseases including lupus are gender biased, with females outnumbering males 9:1 (1–5). Emerging evidence shows that sex hormones influence the expression and function of regulatory cells in both mice and humans (6) and that regulatory T cells (T_regs_), which play an important role in the regulation and maintenance of a normal immune responses, are impaired in numbers or in function in many autoimmune diseases including SLE (7–9). Both CD4^+^ and CD8^+^ T_regs_ in the peripheral immune system have important roles in suppressing autoimmune disease (10–15). Impaired development and function (16–19) or removal (20–23) of T_regs_ can also promote the development of autoimmunity. We have shown previously that T_regs_ suppress autoreactive T and B cells in lupus-prone mice and protect against disease (24–27). Some recent reports suggest a decreased percentage of CD4^+^CD25^+^ cells (28–30); whereas, other reports seem to indicate normal or increased numbers of circulating T_regs_ (31–35) in active SLE patients. These differences may be due to differences in phenotyping methods, analyses, disease status, or therapies. Our own research points to a significantly decreased percentage of T_regs_ in our SLE cohort. However, gender-based differences in the roles of T_regs_, expression of FoxP3, and their regulatory capacities have not been thoroughly studied.

Recent studies have shown that FoxP3 plays a significant role in regulatory T cell differentiation, function, and the prevention of auto-reactivity (8, 36, 37). FoxP3 is a critical transcription factor essential for determining the phenotype, development, and function of T_regs_. FoxP3 deficiency or mutation results in the “Scurfy” phenotype in mice (38) and in humans results in IPEX (Immune dysregulation, Polyendocrinopathy, X-linked) syndrome (8, 9, 39–41).

Sex hormones have been known to play an important role in regulating lupus both in human and animal models (42, 43). Estrogen has been shown to increase calcineurin in T cells of SLE patients but not in age and sex matched healthy controls (44, 45). These previous studies did not examine the role of T_regs_ in SLE patients. A recent study identified the expansion of CD4^+^CD25^+^ and FoxP3^+^ T_regs_ during the follicular phase of the menstrual cycle in healthy females and found that an increase in T_regs_ correlated with changes in serum estradiol levels (46). In healthy males, testosterone antagonists have been shown to cause significant decreases (~30%) in the percentage of CD4^+^CD25^+^ T cells in comparison with baseline and with subjects in the placebo group. This decline normalized with the return of natural hormone levels after the antagonists were discontinued (47). In males with SLE, imbalances in estrogens and androgens could contribute to susceptibility to the disease (48–52).

Testosterone suppresses both IgG anti-dsDNA antibody and total IgG production in PBMCs from SLE patients (53, 54). Low levels of plasma androgens was reported (55) and androgen administration has been demonstrated to improve disease activity (56) in women with active SLE disease. However, this study (56) did not evaluate T_reg_ function or the expression of FoxP3.

In the present study, we provide evidence that sex hormones and gender influence both the number and phenotype of T_regs_ and the T_reg_ expression of FoxP3 differentially in men and women and also in SLE patients and healthy controls. Notably, we have found that plasma levels of estradiol are increased and testosterone levels are decreased in SLE females compared to healthy females. Furthermore, we have found that T_reg_ exposure to testosterone *in vitro* increases FoxP3 expression in SLE females. Finally, we have demonstrated that plasma concentrations of testosterone in SLE females positively correlates with levels of FoxP3 expression. These data suggest that sex hormones and gender play pivotal roles in the regulation and maintenance of immune responses and provide novel evidence for a modulatory role of 17β-estradiol and androgen (DHT) in the phenotype and regulation of immune responses in autoimmunity.

## Materials and Methods

### Subjects

We enrolled 27 subjects who were 18 years or older and fulfilled the American College of Rheumatology revised criteria for the classification of SLE (57, 58) and 23 healthy donors (19–70 years of age) with no history of autoimmune disease. Patients with comorbid conditions were excluded from the study. Disease activity was recorded based on the SLE disease activity index (SLEDAI) (59). For estradiol and testosterone measurement, we obtained control and SLE plasma samples from the UCLA Rheumatology biobank. The study was approved by the Institutional Review Board of the University of California Los Angeles. Written informed consent was obtained from each subject who participated in the study.

### Cell Isolation and Preparation

T cell enriched peripheral blood mononuclear cells (PBMCs) were isolated on a density gradient (Histopaque-1077, Sigma-Aldrich, St. Louis, MO, USA) from blood samples of lupus patients and healthy volunteers. Lymphocytes were washed twice in serum free media. Red blood cells (RBC) were lysed with RBC lysing solution (Sigma-Aldrich, St. Louis, MO, USA). CD4^+^CD25^+hi^CD127^low^ and CD8^+^CD25^+hi^CD127^low^ T_regs_ were sorted after staining using a FACS Aria flow cytometer (BD Biosciences) for sex hormone experiments.

### Immunophenotyping and Flow Cytometry

Peripheral blood mononuclear cells (PBMCs) from patients and healthy volunteers were stained with CD4-FITC, clone (RPA-T4); CD8-PerCP, clone (SK1); CD25-APC, clone (BC96); and CD127-PE, clone (hIL7R-M21) fluorochrome-conjugated monoclonal antibodies (mAb). Intracellular staining for FoxP3 (clone-PCH101) was performed after cell surface staining by fixation and permeabilization as per manufacturer protocol (eBiosciences, San Diego, CA). The antibodies for cell surface staining and isotype controls were from BD Biosciences, eBiosciences, and from BioLegend. (San Diego, CA). Data were collected using FACSCalibur (BD Biosciences) and analyzed by BD Cell Quest software (Becton-Dickinson, Mountain View, CA) or FCS De Nova software (Thornhill, Ontario, Canada).

### Cell Culture

PBMCs and sorted CD4^+^CD25^+hi^CD127^low^ T_regs_ (1-2x10^6^ cells) were cultured with the sex hormones 17β-estradiol (30, 60–100, 500 pg/ml) or testosterone (30, 60, 100, 500 pg/ml), or with TGFβ (20 ng/ml) with and without fetal calf serum in complete media for 24–72 h. After culture, supernatants were obtained; cells were washed and stained for FACS analyses, and lysed for RNA and Western blot analyses.

### ELISA

Estradiol and testosterone levels were measured in plasma and culture supernatants by commercial ELISA (Calbiotech, Inc., Spring Valley, CA) as per manufacturer instructions.

### RNA Isolation and Real Time PCR

RNA was isolated from sorted and cultured T_regs_ with TRIzol (Invitrogen) as per the manufacturer's protocol. Real time PCR was used to analyze mRNA gene expression as described earlier (1, 26, 27). Human FoxP3 specific primers and probes were synthesized from Applied Biosystems. Human FoxP3 primer and probe sequences are as follows: FoxP3 forward, 5′-TCTTCTCGGTATAAAAGCAAAGTTGTT-3′; reverse, 5′-GTGAAGTGGACTGACAGAAAAGGAT-3′; probe, 6*FAM*-TGATACGTGACAGTTTCCCACAAGCCA-*TAMRA*. Human GAPDH primers and probes were obtained from Applied Biosystems. The amplification primers were used at 900 nM and probes at 200 nM. All samples were run in duplicate. Data was normalized with the house-keeping gene GAPDH.

### Statistical Analyses

Data was analyzed using Prism 4.0 (GraphPad Software, San Diego, CA). Comparisons were performed using paired one- or two-tailed test. Results are expressed as mean ± SEM. *p* < 0.05 was considered significant.

## Results

### CD4^+^CD25^+hi^FoxP3^+^ and CD8^+^CD25^+hi^FoxP3^+^ T_regs_ Are Reduced in SLE Patients

**Figure 1:**
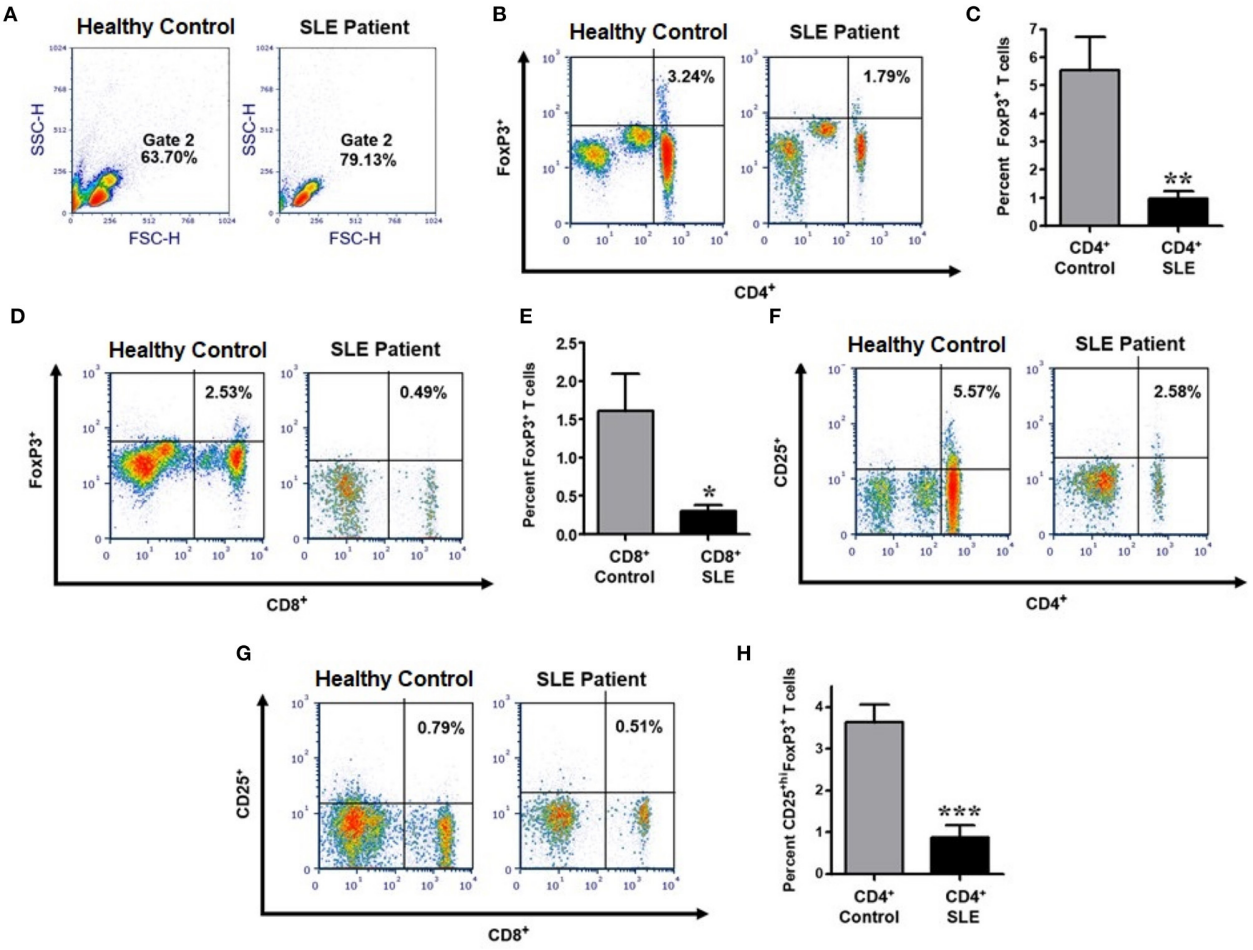
The percentage of CD4^+^ and CD8^+^ expressing characteristics of regulatory T cells are significantly reduced in SLE patients compared to gender and age matched healthy controls. Representative FACS analysis from PBMC of active female SLE patients compared with healthy controls **(A,B,D,F,G)**. Peripheral blood mononuclear cells (PBMC) were isolated from 20 to 30 ml of blood obtained from SLE patients and healthy controls. 10,000 cells were gated and analyzed by FACS. Representative FACS analysis of FoxP3^+^ T cells **(B)** (with percent of positive cells in the Upper-Right quadrant indicated) were analyzed after gating of CD4^+^ T cells and CD8^+^ T cells from PBMC. **(C)** Cumulative data of CD4^+^FoxP3^+^ T cells in healthy controls (*n* = 14) and SLE patients (*n* = 25). **(D)** Representative FACS analysis of CD8^+^FoxP3^+^ T cells from PBMC of SLE patient vs healthy control. **(E)** Cumulative data of CD8^+^FoxP3^+^ T cells in healthy controls (*n* = 13) and SLE patients (*n* = 16). **(F)** Representative FACS analysis of CD4^+^CD25^+^ T cells from SLE patient vs. healthy control. **(G)** Representative FACS analysis of CD8^+^CD25^+^ T cells from SLE patient vs. healthy control. **(H)** Cumulative data of CD4^+^CD25^+hi^FoxP3^+^ T cells in healthy controls (*n* = 10) and SLE patients (*n* = 12). *p* values indicating significant differences are shown in each panel **(C,E,F)**. *p < 0.05, **p < 0.01, ***p < 0.001.

In order to determine the number, phenotype, and homeostatic regulation and effects of sex hormones on T_regs_ in lupus patients compared to healthy controls, we performed an extensive immunophenotyping of PBMCs from each group (see gating scheme Figure 1A). Treg cells were identified as those cells expressing FoxP3 and CD25^+^^high^. We found that lupus patients exhibited significantly reduced percentages of CD4^+^FoxP3^+^ (*p* < 0.0041) and CD8^+^FoxP3^+^ T cells (*p* < 0.0102) when compared to healthy controls (Figures 1A–E). We also characterized levels of CD4^+^CD25^+^ (Figure 1F) and CD8^+^CD25^+^ T cells (Figure 1G) between healthy control and SLE patient and found that the percentage of CD4^+^CD25^+hi^FoxP3^+^ T_regs_ in peripheral blood was also significantly reduced in patients with SLE relative to healthy controls (Figure 1H; *p* < 0.0005). Collectively these data demonstrate that lupus patients have reduced percentages of both CD4^+^ and CD8^+^ T_regs_.

### CD4^+^CD25^+hi^ and CD8^+^CD25^+hi^ T Cells From Healthy Males Have Higher FoxP3 mRNA Levels Than Healthy Females

**Figure 2:**
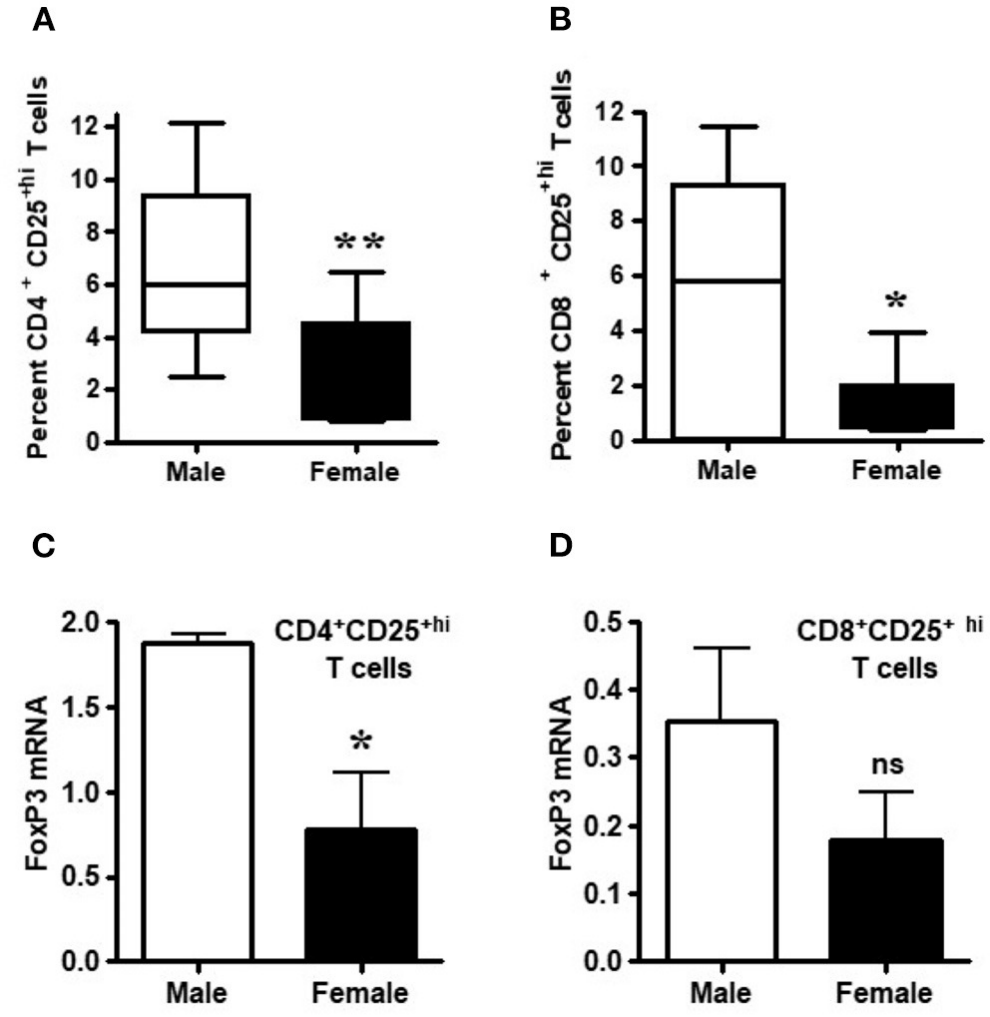
Circulating T_regs_ of healthy males have higher FoxP3 mRNA than cells from healthy females. Numbers of circulating CD4^+^ and CD8^+^ regulatory T cells are decreased in healthy females. **(A)** CD4^+^ regulatory T cell numbers were measured after FACS staining by CD4, CD8, and CD25 monoclonal antibodies from 6 healthy males and 10 healthy females, **p < 0.007 by Mann Whitney two-tailed *t* test. **(B)** CD8^+^ T_regs_ from 8 healthy males and 10 healthy age matched females, *p < 0.02 by paired two-tailed *t* test. Percent positive CD4^+^ T_reg_ and CD8^+^ T_regs_ were determined from total CD4 and CD8 cells from PBMCs. T cells from male and female healthy subjects were sorted by FACS and total RNA isolated from CD4^+^CD25^+hi^ **(C)** and CD8^+^CD25^+hi^ **(D)** cells. 100 ng of RNA from each male and female was used for real-time PCR to quantitate FoxP3 mRNA levels, and data were normalized with GAPDH. Data shown are from 3 males and 5 females. *p < 0.05, **p < 0.001, ns, not significant.

Healthy males had significantly higher percentages of CD4^+^CD25^+hi^ and CD8^+^CD25^+hi^ T_regs_ relative to healthy females (Figures 2A,B; *p* < 0.007 and *p* < 0.02). Since lupus is a gender-biased disease with a female to male ratio of 9:1, we determined whether FoxP3 expression in healthy male and female individuals varies in their regulatory T cell compartments. FoxP3 expression in CD4^+^ and CD8^+^ regulatory subsets, CD4^+^CD25^+hi^ and CD8^+^CD25^+hi^ T cell subsets were sorted by FACS. The sorted cells were subjected to RNA isolation from both healthy male and female subjects. We found that CD4^+^CD25^+hi^ and CD8^+^CD25^+hi^ subsets of healthy males had 2-3 times higher FoxP3 mRNA compared to healthy females (Figures 2C,D). Overall, we found that circulating CD4^+^CD25^+high^ and CD8^+^CD25^+high^ T cells are higher in healthy males than healthy females and, although, FoxP3 expression is decreased in both CD4^+^ and CD8^+^ T cells, it was only significantly decreased within the CD4^+^ T cell compartment.

### Females Have Less Total FoxP3 Message in PBMCs Relative to Males; Evidence Suggests That TGFβ Promotes FoxP3 Expression in Both Sexes

**Figure 3:**
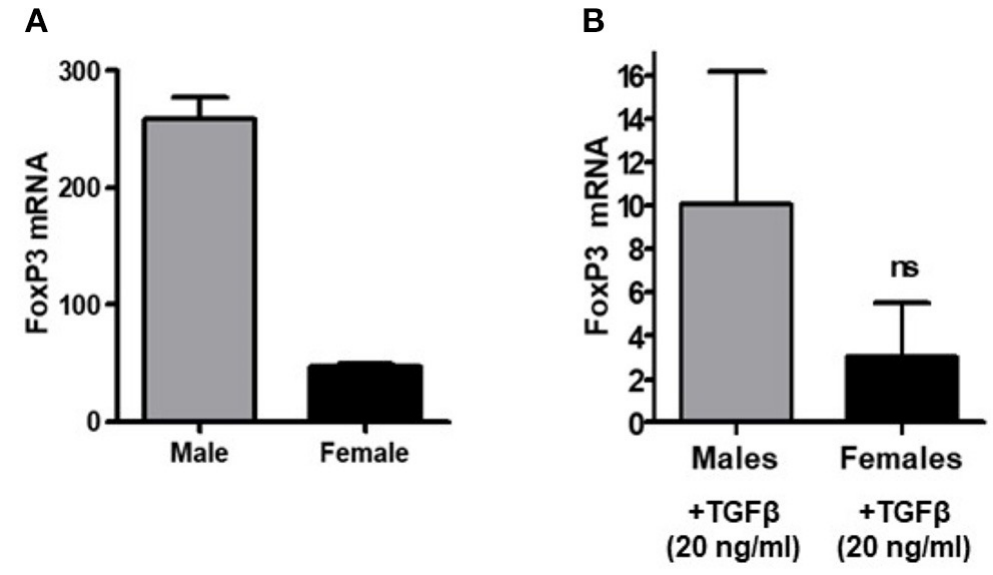
TGFβ promotes FoxP3 expression in human PBMCs *in vitro*. PBMCs (2–4 × 10^6^) were isolated from healthy males (*n* = 5) and healthy females (*n* = 5) and cultured with TGFβ (20 ng/ml) for 24–48 h range in complete media. RNA was isolated and real time PCR was performed for FoxP3 mRNA expression. 100 ng of RNA was used for FoxP3 mRNA expression with a specific primer and probe for the human FoxP3 gene. **(A)** PBMCs were isolated from a 21-year-old male and 22-year-old female, RNA was isolated, and real-time PCR was performed. Experiment was performed in triplicate and error bar shows the values from the triplicates. A deficiency of FoxP3 mRNA levels was noted in the female subject as compared to male. **(B)** PBMCs from healthy males (*n* = 5) had a larger magnitude of response to TGFβ treatment (20 ng/ml) than female cells (*n* = 5).

Having examined the expression of FoxP3 in CD4^+^ and CD8^+^ T_regs_ of healthy males and females, we were interested to see whether transforming growth factor-β (TGFβ) promotes FoxP3 expression differently in whole peripheral blood mononuclear cells (PBMCs) of healthy males and females. Transforming growth factor-β (TGFβ) is a multifunctional cytokine regulating T cell biology (60, 61). It has been shown to induce FoxP3^+^ T regulatory cells from CD4^+^CD25^−^ precursors (12, 62, 63). To address TGFβ effects on FoxP3 expression in healthy males and females, PBMCs were obtained, total RNA isolated, and real-time PCR performed to analyze FoxP3 expression. We found that female have less FoxP3 expression compared to age matched male healthy subject (Figure 3A). Overall, TGFβ increased FoxP3 expression in both healthy male and healthy female PBMCs (Figure 3B) however, the fold increase of FoxP3 expression was less in healthy females than in healthy males. These findings of a lower response to TGFβ in terms of expression of FoxP3—a classical marker of T_regs_–in healthy females compared to healthy males might be one explanation of why SLE and other autoimmune disorders are predominantly expressed in females.

### Female SLE Patients Have Altered Sex Hormone Levels—Increased Plasma Estradiol Levels and Decreased Testosterone Levels

**Figure 4:**
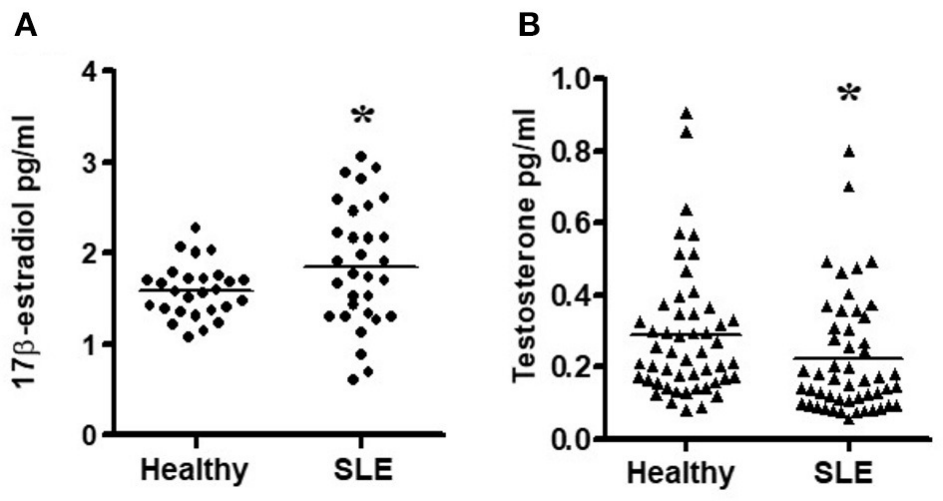
Estradiol and testosterone levels in plasma of SLE patients and healthy controls. **(A)** Female SLE patients (*n* = 31) have increased plasma estradiol levels compared to female healthy controls. (*n* = 27) and **(B)** Female SLE patients (*n* = 51) have decreased testosterone levels compared to healthy female controls (*n* = 51). Plasma estradiol and testosterone levels were measured by ELISA. *p < 0.05.

Since chronic exposure to estradiol leads to activation of pro-inflammatory cells and genes, we measured 17β-estradiol levels in SLE patients and age- and sex-matched controls. We found that female SLE patients have significantly increased plasma estradiol levels compared to healthy controls (Figure 4A). We also found that testosterone levels were decreased in female SLE patients compared to healthy female controls (Figure 4B).

### Sex Hormones Influence CD4, CD25, and FoxP3 Expression Differentially in Healthy Males and Females and in SLE Female PBMCs

**Figure 5:**
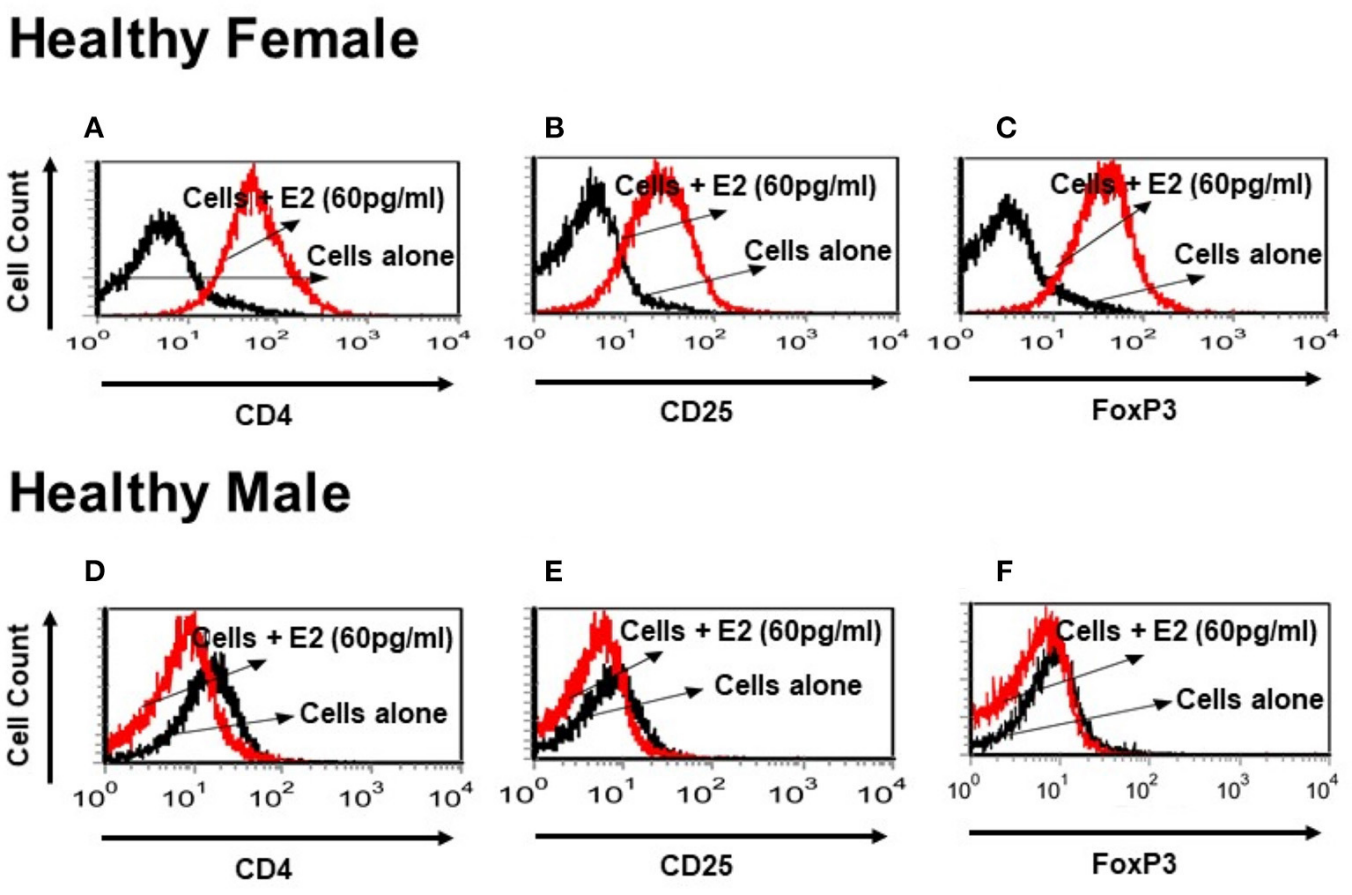
Estrogen increases expression of CD4, CD25, and FoxP3 *in vitro* in a healthy female subject but not in a healthy male subject. PBMCs (2–4 × 10^6^) were isolated from a healthy female and a healthy male subject and cultured for 24–48 h range with physiological concentrations of E2 (60 pg/ml). Estrogen increases CD4 and CD25 cell surface expression and intracellular FoxP3 expression in a healthy female PBMCs **(A–C)**. These effects were not seen in a healthy male PBMCs cells **(D–F)**.

**Figure 6:**
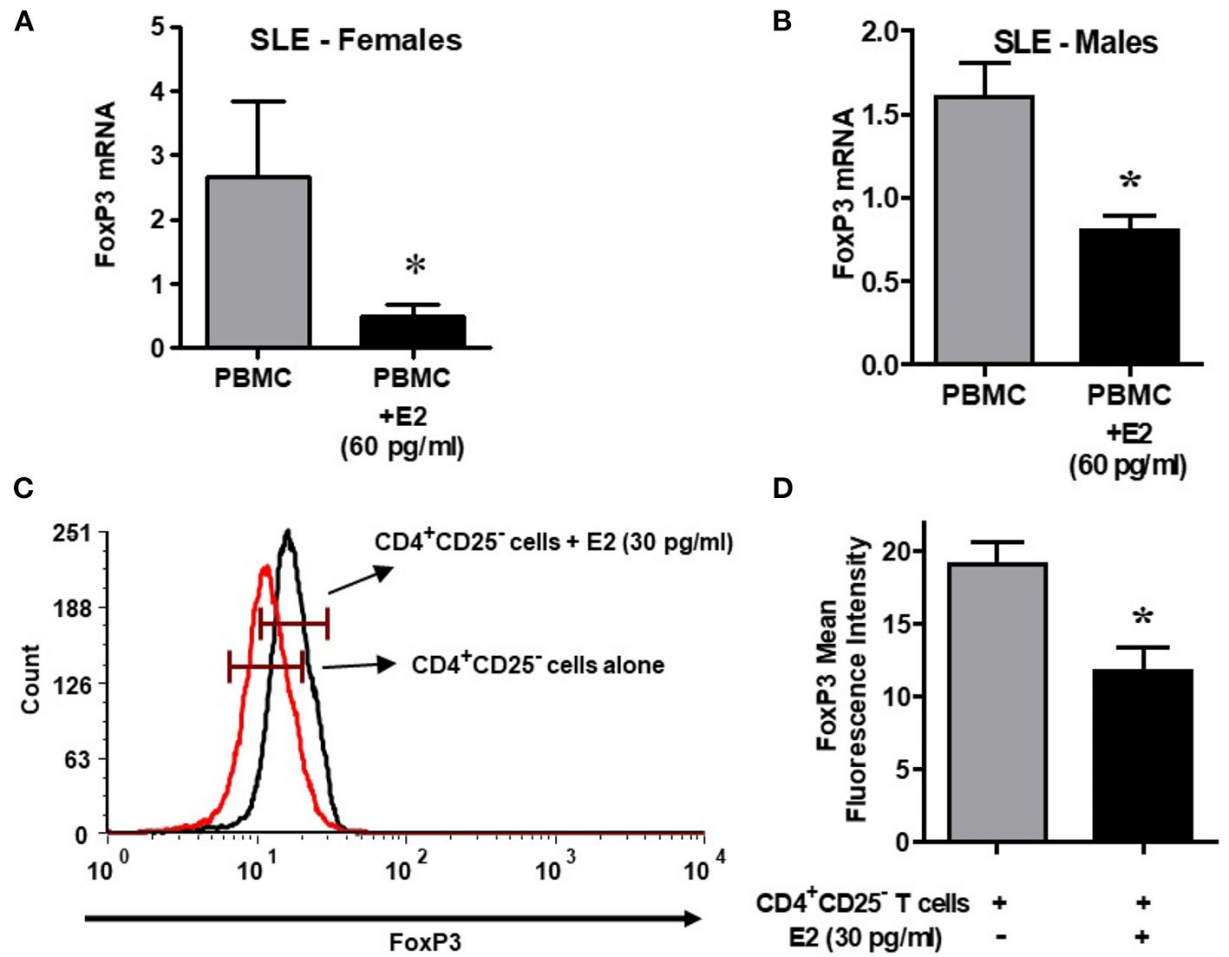
Lupus patient immune cells respond to E2 with a decrease in FoxP3 expression. Estrogen decreases expression of FoxP3 in both female and male SLE patient PBMCs. Lupus patient PBMCs (2–4 × 10^6^ cells) were isolated and cultured (24–48 h range) with E2 at physiological concentrations (60 pg/ml). RNA was isolated, and real time PCR was performed with FoxP3 primers and probes. GAPDH was used as the house keeping gene. FoxP3 mRNA expression was reduced in female (*n* = 5) and male (*n* = 3) SLE pts **(A,B)**. **(C)** Estrogen decreases FoxP3 protein in CD4^+^CD25^−^ T cells from SLE patients. CD4^+^CD25^−^ T cells were isolated from female SLE patient cells (*n* = 3) and cultured for in the 24–48 h range with E2 at 30 pg/ml concentration. FoxP3 intracellular protein was measured by FACS. **(D)** Mean fluorescence intensity (MFI) decreases in CD4^+^CD25^−^ T cells from SLE patients treated with E2. *p < 0.05.

It is not clear whether sex hormones influence CD4, CD25 and FoxP3 expression differentially in humans, both healthy and in SLE patient cells. To address this, we isolated PBMCs from healthy male and female volunteers and cultured their PBMCs with different concentrations of 17β-estradiol (E2). In preliminary *in vitro* experiments using different concentrations of E2 (30–150 pg/ml), we determined that maximal responses in both healthy and SLE individuals occurred at the 30–60 pg/mL range (data not shown). Of significance, we found that incubation with E2 at physiologic range (60 pg/ml) significantly increased CD4, CD25, and FoxP3 (mean fluorescence intensity) expression in PBMCs from a healthy female (Figures 5A–C) but not in PBMCs from a healthy male (Figures 5D–F). In contrast, in SLE patients (both males and females) PBMCs treated with E2 at a physiological dose (60 pg/ml) was associated with significantly reduced FoxP3 mRNA expression (Figures 6A,B). These differences in response to E2 in SLE patients' immune cells vs. healthy cells suggest that the disease-inflammatory microenvironment may play a significant role. Next, we wanted to see how CD4^+^CD25^−^ T cells from SLE patients would response to E2. We found that CD4^+^CD25^−^ T cells treated with E2 were unable to drive FoxP3 protein expression (Figure 6C) and that the mean fluorescence intensity of FoxP3 was significantly (*p* < 0.04) decreased in E2-treated CD4^+^CD25^−^ T cells (Figure 6D). These data suggest that in healthy females E2 promotes an increase in regulatory T cell numbers and FoxP3 expression; whereas, females with SLE have defective regulatory T cell responses to E2 at physiologic levels. Therefore, females with SLE do not expand T_regs_ normally in response to estradiol stimulation.

### Androgen/Testosterone Increases the Expression of FoxP3 mRNA and Protein in Regulatory T Cells of SLE Patients

**Figure 7:**
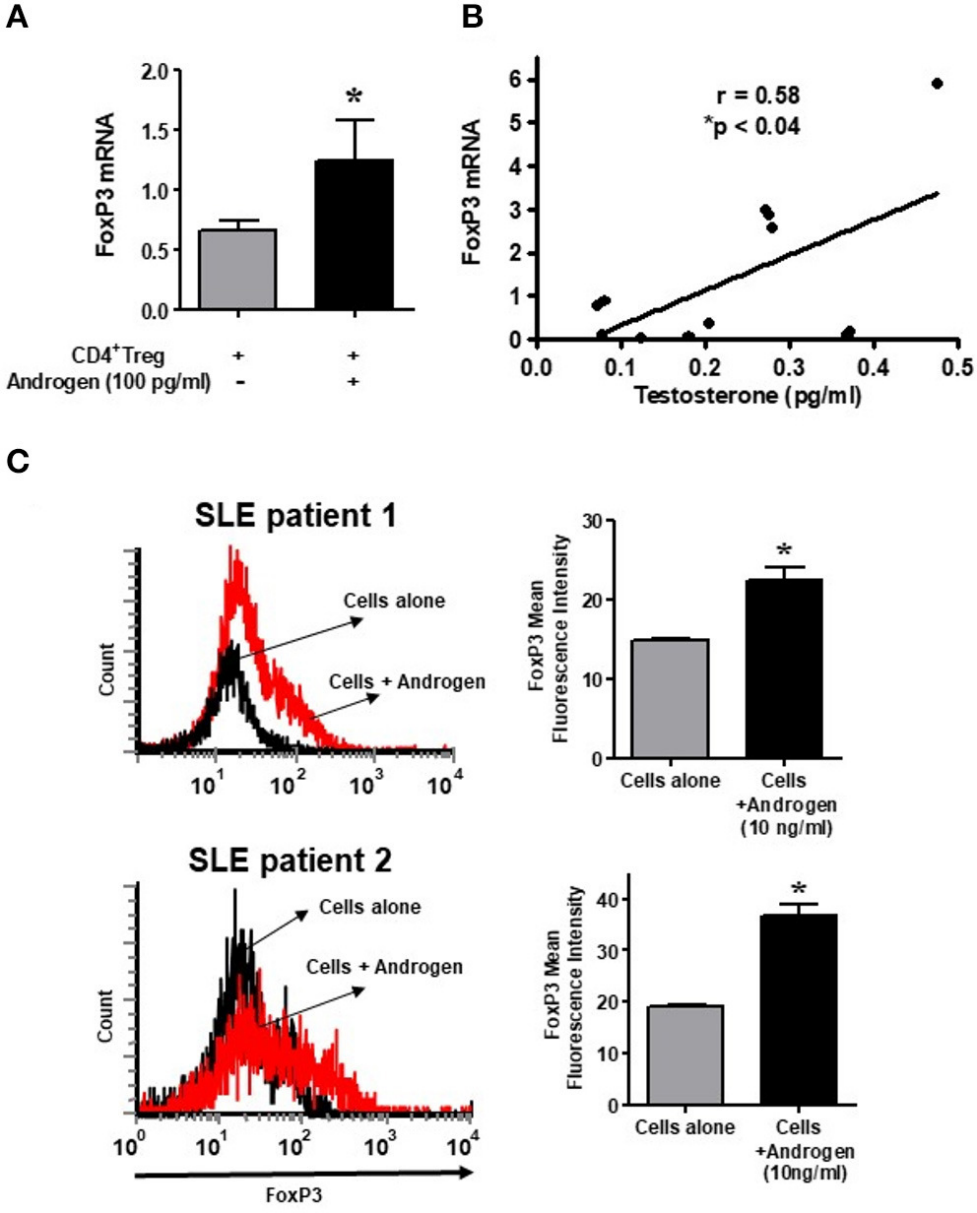
Testosterone increases the expression of FoxP3 mRNA in female SLE patient T_regs_ *in vitro*, and the plasma level of testosterone correlates with FoxP3 mRNA in SLE patients. **(A)** Androgen/testosterone treatment (100 pg/ml) increases FoxP3 expression significantly in female SLE patient' T_regs_. CD4^+^CD25^+high^CD127^low^ T_regs_ were isolated from female SLE patients (*n* = 7-10) cells after cultured for 24–48 h range with androgen. RNA was isolated, and real time PCR was performed with FoxP3 primers and probes. GAPDH was used to normalize data. **(B)** Positive correlation between plasma levels of testosterone and expression of FoxP3 in female SLE patients (*n* = 12) immune cells. *p < 0.05. **(C)** Female PBMCs were isolated from SLE patients and cultured at a density of 2-3 × 10^6^ cells per well with and without androgen (10 ng/ml) for 24–48 h range. Cells were washed, stained with CD4 and FoxP3 antibodies, and analyzed with FACS. A minimum of 10,000 cells were gated. Representative FACS analysis from two female SLE patients is shown. Mean fluorescence intensity (MFI). *p < 0.05.

In the present study, we also tested the androgen effect on isolated CD4^+^CD25^+^CD127^low^ T_regs_ in female SLE patients. Isolated cells were cultured with testosterone (100 ng/ml) and then lysed, RNA isolated, and RT-PCR performed. We found that treatment with testosterone/DHT (100 ng/mL) significantly increased FoxP3 mRNA expression in SLE patient' CD4^+^ T_regs_ (Figure 7A). The positive effect of testosterone on FoxP3 expression is further suggested by the data shown in Figure 7B, which indicates that plasma concentrations of testosterone in females with SLE correlates significantly with the expression of FoxP3 in their CD4^+^CD25^+^CD127^low^ T cells. Furthermore, we found that incubation with androgen increased the expression of total FoxP3 protein in the PBMCs of female SLE patients (Figure 7C). These data suggest that androgens positively regulate FoxP3 expression in SLE patients.

## Discussion

Environmental factors, genetic defects, and hormones can regulate immune responses and therefore may influence SLE susceptibility (64–67). Regulatory T cells (T_regs_) play a key role in maintaining immune homeostasis including the prevention of autoimmunity, maintenance of self-tolerance, and regulation of immune responses against infection (68–70). The functional failure of T_regs_ can result in development of autoimmune diseases including SLE (18, 71–73). Regulatory B cells (B_reg_) and myeloid-derived suppressor cells (MDSC) (74–79) and type1 regulatory T cells (CD4^+^IL-10^+^FoxP3^−^) (80– 85) have an immunosuppression role and promote immune tolerance. Genetic polymorphisms in the *FoxP3* gene and imbalances of regulatory T cells and autoimmunity have also been reported (86) as-well-as polymorphisms of genes involved in T_regs_ activation and function (87). Gender effects have also been reported.

Women are more prone than men to the development of autoimmune diseases including SLE (3, 4) and the role of sex hormones (17β-estradiol and androgen) has been demonstrated in SLE (88). The female sex hormone (estrogen) contributes to the pathogenesis of SLE by activating T cells and by modulating the function of regulatory T cells (89–91). However, it is not clear whether sex hormones and/or gender regulate the function of these regulatory cells or expression of markers including FoxP3 differentially in humans.

The role of sex hormones (17β-estradiol and androgen) has been demonstrated in SLE (88). The female sex hormone (estrogen) contributes to the pathogenesis of SLE by activating T cells and by modulating the function of regulatory T cells (89–91). In addition, an association of single-nucleotide polymorphisms in the *FoxP3* gene have been correlated with SLE susceptibility (92). However, it is not clear whether sex hormones and/or gender regulate the function of these regulatory cells or expression of markers including FoxP3 differentially in humans. In the present study, we did not address the issue of gene polymorphisms or the possibility that individual SNPs may play a significant role in healthy T_reg_ populations which have low FoxP3 expression. Future study will be needed to address these possibilities. Previous studies have shown that estradiol treatment of PBMCs affects T cells, B cells, and monocytes (54, 93, 94); and gender differences in estrogen receptor (ER) expression were documented (95). However, the effect of gender and sex hormones on the phenotype and function of T_regs_ and expression of FoxP3 in SLE patients compared to healthy controls is less well studied. Recent evidence in a mouse model of autoimmune diseases indicates that female sex hormone (17β-estradiol-E2) influences the expression of FoxP3 and T_reg_ number and function. Further, estradiol has been shown to influence the activation and function of many immune cells (74, 93) including CD4^+^ (Th1, Th2, Th17, and T_reg_) and CD8^+^ T cells (96–98). It has been reported that estrogen exposure directly activated T cells through the cell membrane estrogen receptor (99) and that 17β-estradiol receptors are differential expressed in women with SLE (100).

Estrogen binds to nuclear receptors (ERα and ERβ) on various cells, including CD4^+^ T cells, thus altering the rate of gene transcription (48-51). It also acts independently of ER (estrogen receptor) through alterations in the plasma membrane. Exposure of T cells to 17β-estradiol stimulates kinase activation and calcium flux. In normal mice, administration of physiologic doses of 17β-estradiol to ovariectomized females increased 2-to-3-fold the numbers of CD4^+^CD25^+^ and CD4^+^FoxP3^+^ T cells in PBL, spleens and lymph nodes, suggesting that 17β-estradiol in the absence of other ovarian hormones strongly influences expansion of T_regs_ (101). *In vivo* or *in vitro* exposure to 17β-estradiol increases CD4^+^CD25^+^ T cell numbers and FoxP3 expression in Experimental Autoimmune Encephalomyelitis (EAE) (102).

Male hormones (androgens) also influence the immune response in many diseases including SLE (47). In males with SLE, imbalances in both estrogens and androgens could contribute to increased susceptibility to the active disease (48–52). Earlier studies indicated that testosterone suppressed both IgG anti-dsDNA antibody and total IgG production in PBMCs from SLE patients (53, 54). Androgen administration (of prasterone, which can be metabolized to testosterone) has recently been demonstrated to improve disease activity in females with SLE (56). The effect of testosterone on CD4^+^ T_regs_ and FoxP3 expression, stability, and plasticity in SLE patients is not clear and has not been studied extensively. Although recent evidence suggests that FoxP3 protein stability is controlled by several proteins including cyclin dependent kinase−2 (CDK-2) (103), Pim-2 kinase (104), Nemo-like kinase, and CNS2 (non–coding sequence 2 demethylation by TET (ten-eleven translocation) protein (105, 106). However, the molecular mechanisms that control FoxP3 stability and T_reg_ plasticity remains to be identified in SLE.

In the present study, we demonstrate that the percentage of CD4^+^ and CD8^+^ T_regs_ are significantly reduced in SLE patients compared to gender and age matched healthy controls (Figure 1); and that the percentage of both CD4^+^ and CD8^+^ T_regs_ is reduced in healthy females compared to healthy males. Our data is the first report that healthy male cells express higher FoxP3 mRNA levels than healthy female cells (Figure 2). We show that female SLE patients have increased plasma levels of estradiol (Figure 4) and that incubation of CD4^+^ T_regs_ with 17β-estradiol (at physiological levels) either maintains or decreases FoxP3 expression in females with SLE, in contrast to inducing a significant increase in CD4^+^ T_reg_ in healthy females (Figures 5, 6). We further show that TGFβ treatment induces FoxP3 expression in PBMC of both healthy males and females, but at a larger degree in males than healthy females (Figure 3). At the mechanistic level, we demonstrate that estrogen increases expression of CD4, CD25, and FoxP3 in a healthy female but not in a healthy male subject (Figure 5). Finally, at the clinical and translational significance level, we showed for the first time that in female SLE patients (1) testosterone levels are reduced (Figure 4); (2) testosterone exposure increases the expression of FoxP3 mRNA in T_regs_ (Figure 7); and (3) that the plasma levels of testosterone positively correlates with FoxP3 mRNA (Figure 7). Thus, our data suggest that sex hormones and gender influences the expression of FoxP3 and regulatory T cells differentially in SLE.

We postulate that women are predisposed to SLE and to the maintenance of disease activity in part because of their relatively high levels of estradiol (E2), and their low levels of testosterone and related active metabolites. We also suggest that the sex hormone (estradiol that has higher concentrations in females) work in part by suppressing the numbers and functional activity of CD4^+^ T_regs_, with resultant inadequate suppression of CD4^+^CD25^−^ effector T cells and of autoantibody-producing B cells. Our data suggest that CD4^+^CD25^+hi^ and CD4^+^FoxP3^+^ T cells are lower in numbers in women with SLE than in matched healthy controls, and that these cells are relatively insensitive to E2 stimulation at the levels that increase FoxP3 expression in normal T_regs_ but not in SLE. Our results are in-line with other investigators who found that estradiol treatment enhances increased expression of CD25^+^ T cells and increased FoxP3 expression in mice treated with estradiol (102). Furthermore, estradiol treatment increased T_reg_ numbers and functions and induces FoxP3 expression both *in vitro* and *in vivo* (6). These data indicate that lupus patient' immune cells (PBMCs) behave differently than those from healthy subjects when they are cultured *in vitro* with 17β-estradiol (Figure 6).

Androgen-induced immunosuppression was reviewed recently (107) in both sexes and shown to affect the differentiation and function of T_regs_ differently in men and women. *In vivo* administration of androgens to women with adrenal insufficiency and rats with experimental autoimmune orchitis has been shown to increase T_reg_ numbers (108, 109). Further it was suggested that, androgens are capable of directly converting peripheral T cells into T_regs_ in women. However, in a contradicting report it was suggested that androgens interfere with T_reg_ function in men, as occurs in a mouse model of Sjögren's syndrome which predominantly affects male mice (110). Further it has been shown that administration of 5-dehydroepiandrosterone (DHEA), which is metabolized to testosterone, reduces disease activity in women with SLE (56). However, this DHEA study did not evaluate the expression of FoxP3 nor regulatory T cell numbers or function. In our current study, testosterone significantly increases FoxP3 expression in CD4^+^CD25^+hi^ cells from females with SLE *in vitro*. Furthermore, we showed that testosterone increases the expression of FoxP3 mRNA in female SLE patient' T_regs_ (Figure 7A). In addition, we showed that plasma concentrations of testosterone positively correlated in those females with the expression of FoxP3 in their CD4^+^CD25^+hi^ T cells (Figure 7B) suggesting that this response to testosterone may be normal in women with SLE, in contrast to their response to estradiol.

Our data is in agreement with a previous study which showed that androgen causes expansion of T_regs_ and a significant androgen-dependent increase of FoxP3 expression in human T-cells from women; however, this response was not seen in males. The study also identified a functional androgen response element (ARE) within the FoxP3 locus (111) and showed that binding of the androgen receptor (AR) to the ARE leads to epigenetic changes. The authors were able to show that the FoxP3 gene is more responsive to androgen treatment in T cells isolated from women than in men, indicating gender-specific androgen signaling. These studies, together with our data, demonstrate that healthy females may be more susceptible than males to SLE and other autoimmune diseases in part because females have fewer T_regs_ with reduced FoxP3 expression within those cells. In addition, females with SLE have less ability to generate CD4^+^ T_regs_ in response to physiologic concentrations of 17β-estradiol in comparison to healthy females; whereas, an active testosterone metabolite can increase the generation of CD4^+^ T_regs_ in SLE females. The responsiveness of female T cells to induce or transform into T_regs_, under the effect of androgen, may provide a mechanistic basis to control excessive and damaging immune responses (autoimmunity). Future studies will be required addressing the exact mechanism of this immune homeostasis.

In summary, our results provide novel evidence for a functional modulatory role of sex hormones (estradiol and androgen) in the differentiation of T_reg_ cells in SLE. Further, we provided evidence that androgen effects the regulation of the FoxP3 expression on T regulatory cells differentially in women and men.

## Data Availability Statement

The raw data supporting the conclusions of this article will be made available by the authors, without undue reservation.

## Ethics Statement

The studies involving human participants were reviewed and approved by UCLA IRB. The patients/participants provided their written informed consent to participate in this study.

## Author Contributions

RPS conceived the idea, designed the experiments, performed the experiments, obtained data, analyzed, wrote the manuscript, and obtained funding. DSB analyzed the data, figure drawing and edited the manuscript. Both authors contributed to the article and approved the submitted version.

## Conflict of Interest

The authors declare that the research was conducted in the absence of any commercial or financial relationships that could be construed as a potential conflict of interest.

## Abbreviations

E2: 17β-estradiol
T_regs_: regulatory T cells
RT-PCR: real-time PCR
SLE: systemic lupus erythematosus
PBMC: peripheral blood mononuclear cells
TGF-β: transforming growth factor beta
CD25: IL-2 receptor α chain
FoxP3: Forkhead box protein-3
FACS: fluorescence-activated cell sorting.
